# Supplementary material for: Novel Putative Tymoviridae-like Virus Isolated from Culex Mosquitoes in Colombia
Source: Viruses. 2023 Apr 13;15(4):953. doi: 10.3390/v15040953 (PMC10143313; doi:10.3390/v15040953)
Supplement: Supplementary file 1 [file viruses-15-00953-s001.zip › Supplementary figure legends.pdf]

## Supplementary figure legends

**Figure S1**. Functional domains predicted for the coding regions of the putative *Guachaca virus* (GUAV). (a) ORF1: The Methyltransferase, Peptidase, Helicase, and RNA-dependent RNA Polymerase domains were predicted from the primary sequence. (b) Coat: A signal cleave region was predicted near the amino-terminal region of the protein, followed by several antigenic domains through the soluble portion of the protein.

**Figure S2**. Growth curve of the putative GUAV in C6/36 cells through RT-qPCR. CT values ± standard deviations were estimated for triplicates every experimental point during the time course.

**Figure S3**. Growth curve of the putative GUAV in human cells through RT-qPCR. CT values ± standard deviations were estimated for every experimental point during the time course of 5 to 13 days post-infection in HEK 293, HeLa, A549, and U937 cells. Undetermined CT values representing absence of specific genome detection were arbitrarily assigned a value of zero.
