# Supplementary figures and images for: Novel Putative Tymoviridae-like Virus Isolated from Culex Mosquitoes in Colombia
Source: Viruses. 2023 Apr 13;15(4):953. doi: 10.3390/v15040953 (PMC10143313; doi:10.3390/v15040953)

a)

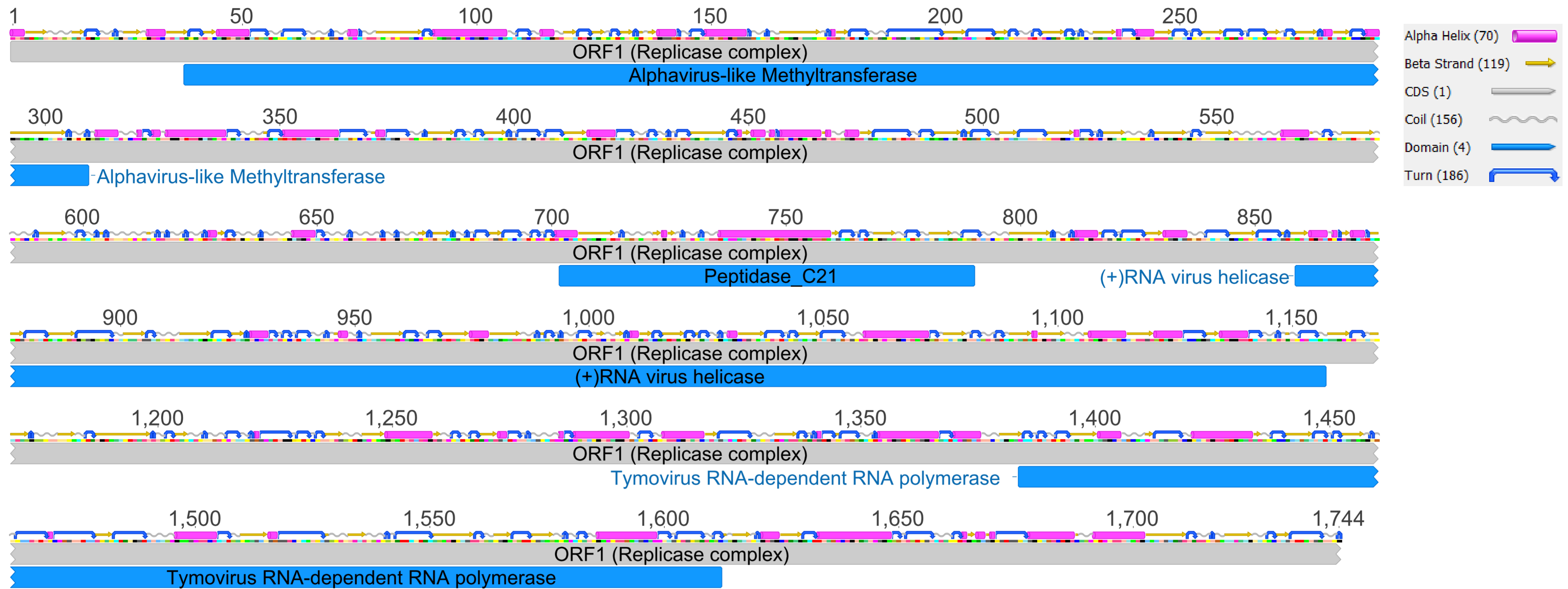

b)

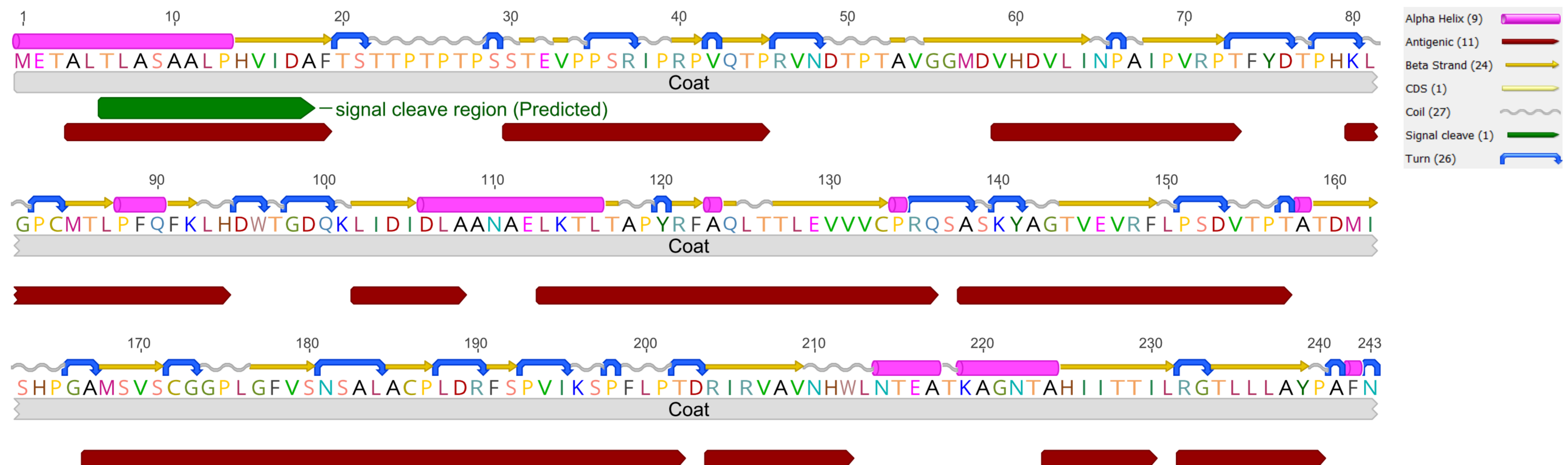

Supplement: Supplementary file 1 [file viruses-15-00953-s001.zip › Supplementary Figure 1.pdf]

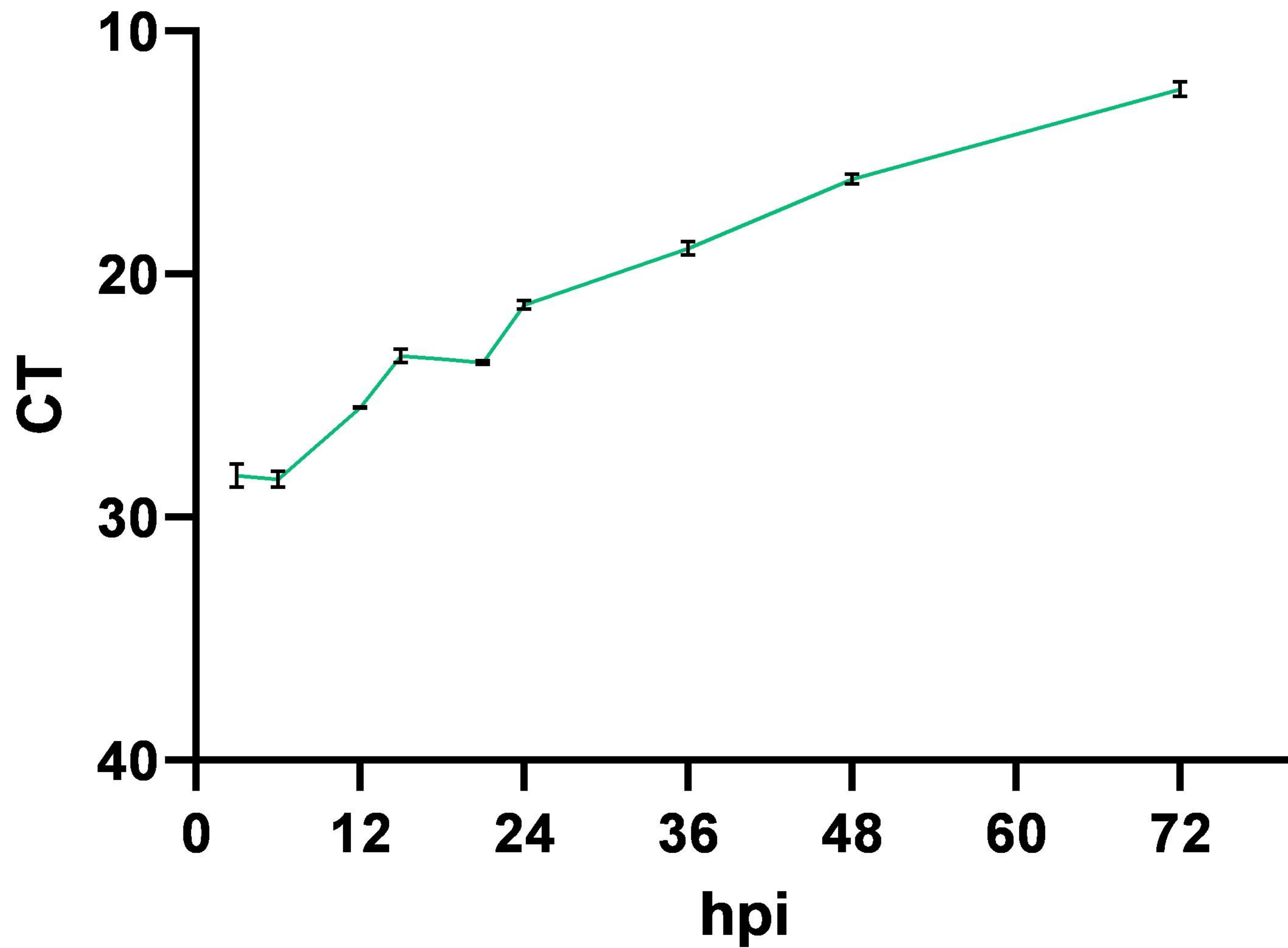

Supplement: Supplementary file 1 [file viruses-15-00953-s001.zip › Supplementary Figure 2.pdf]

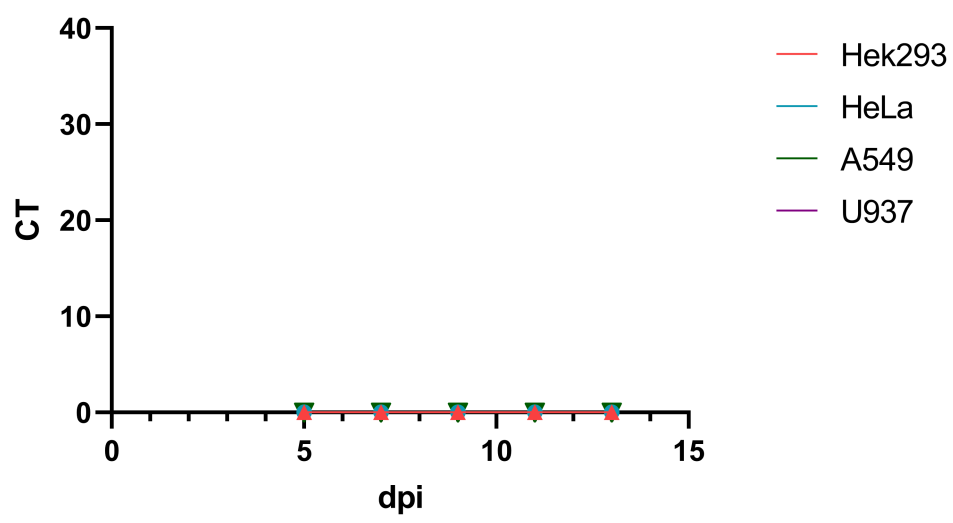

Supplement: Supplementary file 1 [file viruses-15-00953-s001.zip › Supplementary Figure 3.pdf]
